# Triple-Negative Breast Cancer: Molecular Particularities Still a Challenge

**DOI:** 10.3390/diagnostics14171875

**Published:** 2024-08-27

**Authors:** Vlad Bogdan Varzaru, Tania Vlad, Roxana Popescu, Cristian Sebastian Vlad, Aurica Elisabeta Moatar, Ionut Marcel Cobec

**Affiliations:** 1 Doctoral School, Faculty of Medicine, “Victor Babes” University of Medicine and Pharmacy Timisoara, 300041 Timisoara, Romania; 2 ANAPATMOL Research Center, Faculty of Medicine, “Victor Babes” University of Medicine and Pharmacy Timisoara, 300041 Timisoara, Romania; 3 Faculty of Medicine, “Victor Babes” University of Medicine and Pharmacy Timisoara, 300041 Timisoara, Romania; 4 Emergency County Clinical Hospital Pius Brinzeu Timisoara, 300723 Timisoara, Romania; 5 Department of Pharmacology, Faculty of Medicine, “Victor Babes” University of Medicine and Pharmacy Timisoara, 300041 Timisoara, Romania; 6 Clinic of Internal Medicine-Cardiology, Klinikum Freudenstadt, 72250 Freudenstadt, Germany; 7 Clinic of Obstetrics and Gynecology, Klinikum Freudenstadt, 72250 Freudenstadt, Germany

**Keywords:** triple-negative breast cancer, molecular markers, genomic profile

## Abstract

Worldwide, breast cancer (BC) is one of the most common cancers in women and is responsible for the highest number of cancer-related deaths among women, with a special clinical behavior and therapy response. Triple-negative breast cancer (TNBC) is seen as a highly invasive BC, characterized by a short survival, higher mortality, recurrence, and metastasis when it is compared to the other BC subtypes. The molecular subtyping of TNBC based on mRNA expression levels does not accurately reflect protein expression levels, which impacts targeted therapy effectiveness and prognostic predictions. Most TNBC cases exhibit a high frequency of homologous recombination (HR) DNA repair deficiency (HRD) signatures and are associated with a complex genomic profile. Biomarker research in TNBC includes investigating genetic mutations, gene expression patterns, immune system-related markers, and other factors that can provide valuable information for diagnosis, treatment selection, and patient outcomes. Additionally, these biomarkers are often crucial in the development of personalized and precision medicine approaches, where treatments are customized to each patient’s unique characteristics. This ongoing research is essential for improving the management and outcomes of TNBC, which is a challenging and heterogeneous form of breast cancer. The findings of this research have practical implications for refining treatment strategies, particularly in selecting appropriate systemic therapies and integrating traditional treatment modalities like surgery and radiotherapy into comprehensive care plans for TNBC patients.

## 1. Introduction

Worldwide, breast cancer (BC) is one of the most common cancers in women and is responsible for the highest number of cancer-related deaths among women, with a special clinical behavior and therapy response [1,2,3,4,5].

Breast cancer (BC) exhibits a heterogeneous molecular pattern, as defined by the 2013 St. Gallen International Breast Cancer Conference. The molecular subtypes are categorized as follows: luminal A (ER/PR+, HER2−, Ki67+ <  20%), luminal B (ER/PR+ <  20%, HER2−, Ki67+ ≥  20%), HER2+ B2 (ER/PR+, HER2 overexpression), HER2 overexpression (ER−, PR−, HER2 overexpression), basal-like triple-negative BC (TNBC) (ER−, PR−, HER2−), and other special subtypes. The percentages indicate immunohistochemical staining results for patient samples [6]. TNBC, as a subtype of basal-like BC, shows an overlapping gene expression profile in 60–90% [7,8]. TNBC is seen as a highly invasive BC, characterized by a short survival, higher mortality, recurrence, and metastasis when it is compared to the other BC subtypes [3,9,10].

The standard breast cancer management in women includes neoadjuvant chemotherapy, which often involves targeted agents, and conservative surgery, followed by adjuvant radiotherapy with or without adjuvant chemotherapy and/or endocrine therapy. Radiotherapy decreases the rate of local recurrence, and thereby, specific mortality [11]. The surgical therapy standard in breast cancer is represented by total mastectomy or breast-conserving surgery [12,13]. Sentinel lymph node biopsy and/or axillary lymph node dissection are conducted based on clinical guidelines for managing the axilla in breast cancer patients, though these guidelines are still evolving [13,14].

Chemotherapy is the main systemic treatment of TNBC; it shows no response to endocrine therapy [15].

**Figure 1:**
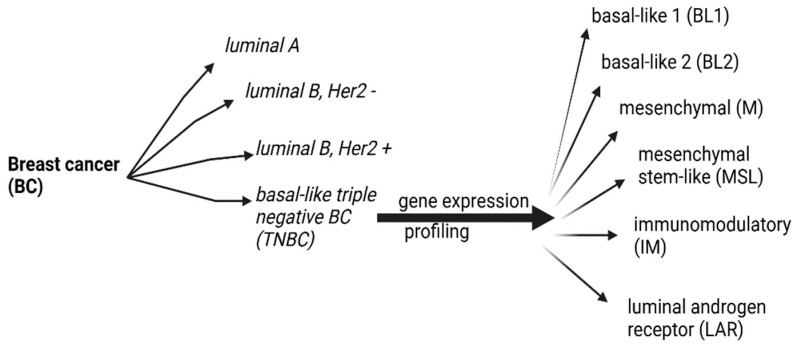
BC molecular subtypes and TNBC gene expression profiling.

Gene expression profiling of TNBC categorizes it into six subtypes: basal-like 1 (BL1), basal-like 2 (BL2), mesenchymal (M), mesenchymal stem-like (MSL), immunomodulatory (IM), and luminal androgen receptor (LAR) (Figure 1) [4,16].

It is unclear if TNBC molecular subtypes are linked to differences in clinical outcomes across races and ethnicities. However, Hispanic and African-American women have a higher proportion of TNBC cases compared to Caucasian women [17].

TNBC molecular subtyping based on mRNA expression levels does not accurately reflect protein expression levels, affecting the efficacy of targeted therapies and the accuracy of prognostic predictions [3].

## 2. Relevant Sections and Discussions

### Genomic Profile and Molecular Markers in the TNBC

More than 85% of TNBCs develop in the context of BRCA1 germline pathogenic variant carriers and 11% to 19% develop in the context of BRCA1 germline or somatic mutations [18]. Mutations in the BRCA1 and BRCA2 genes significantly increase the risk of breast and ovarian cancers, with ovarian cancer typically associated with a poor prognosis [18,19,20]. The BRCA1 and BRCA2 genes are involved in DNA repair. Mutations in these genes are not only risk factors for developing breast cancer but also prevalent in TNBC. Patients with TNBC and BRCA mutations may benefit from PARP inhibitors, a class of drugs that target cancer cells with DNA repair defects [21,22].

Although TNBC encompasses a variety of histologic types of breast cancer, most cases are high-grade invasive ductal carcinomas of no special type [23,24,25].

Carcinomas with apocrine differentiation exhibit a distinct set of somatic alterations, including mutations in PIK3CA, PIK3R1, and AKT1, indicating that activation of the PI3K pathway plays a crucial role in the molecular pathogenesis of the disease [26].

The majority of TNBC cases exhibit a high frequency of homologous recombination (HR) DNA repair deficiency (HRD)-related signatures and are associated with a complex genomic profile in the basal-like immune-activated TNBC subtype [27]. Advances in research have refined and expanded our understanding of TNBC subtypes, leading to the identification of basal-like immune-suppressed (BLIS) and basal-like immune-activated (BLIA) subtypes. These subtypes include many basal-like tumors with germline and/or somatic BRCA1 mutations [27,28,29].

The BLIS subtype is defined by a specific immune phenotype where the immune response is suppressed, contrasting with other TNBC subtypes that might have an activated immune environment [28]. This immune suppression can make the BLIS subtype less responsive to immunotherapy strategies that rely on an active immune response to target cancer cells. Recent research emphasizes the modulatory role of microRNAs in TNBC, especially in the basal-like phenotype. These small non-coding RNAs can regulate gene expression and are implicated in the immune suppression observed in BLIS TNBC [30]. Understanding these regulatory mechanisms may open new avenues for targeted therapies.

The immune-suppressed nature of BLIS TNBC is linked with the expression of immune checkpoint molecules, which can inhibit the activation of T-cells against tumor cells. This inhibition contributes to the resistance of BLIS TNBC to certain therapies, including immunotherapies. Despite the challenges in treating BLIS TNBC, the detailed understanding of its immune suppression and molecular characteristics offers potential targets for therapy. For example, targeting the pathways responsible for microRNA regulation and immune checkpoint activation could provide new therapeutic strategies [31].

The immune-suppressed nature of BLIS TNBC generally indicates a poorer prognosis compared to other TNBC subtypes. This is due to the cancer’s ability to evade immune detection and the challenges in effectively targeting this subtype with current therapies. However, research into the molecular underpinnings of BLIS TNBC holds promise for developing more effective treatments in the future [32].

The basal-like 1 (BL1) subtype is distinguished by its expression of genes that regulate the cell cycle and respond to DNA damage. This subtype is often more proliferative, meaning the breast cancer cells tend to divide and grow rapidly. BL1 tumors may have a higher response to certain chemotherapy regimens that target rapidly dividing cells. Due to their rapid division and growth, BL1 tumors may respond better to certain chemotherapy regimens, especially those therapy regimens targeting rapidly dividing cells. Chemotherapy works by killing or slowing the growth of rapidly dividing cells, both cancerous and normal cells. Since BL1 tumors consist of cells that divide quickly, they may be more susceptible to the effects of these treatments. The effectiveness of chemotherapy in treating BL1 tumors suggests that these treatments can specifically exploit the tumor’s rapid proliferation rate [33,34].

Basal-like 2 (BL 2) is associated with immune system-related gene expression, often showing signs of immune cell infiltration within the tumor microenvironment. This subtype may have a more pronounced immune response [16]. Given the pronounced immune response and the infiltration of immune cells within the tumor microenvironment, BL2 tumors may exhibit a higher sensitivity to immunotherapy approaches. Immunotherapies, such as immune checkpoint inhibitors, work by enhancing the body’s natural immune response against cancer cells.

Checkpoint inhibitors work by targeting proteins such as PD-1/PD-L1 and CTLA-4, which are utilized by cancer cells to avoid detection by the immune system. By blocking these inhibitory signals, immunotherapies can potentially reinvigorate the immune system’s ability to fight the cancer. BL2′s immune-active profile suggests it could be more amenable to such treatments compared to more immune-evasive subtypes [35].

Breast cancer stem cell (BCSC) populations are more prevalent in TNBC, particularly in the BL2 and M subtypes, where mesenchymal-like BCSCs were predominantly found. On the other hand, epithelial-like BCSCs were also identified in the BL1 and LAR TNBC subtypes. This distinction suggests that different TNBC subtypes harbor unique BCSC populations, which could contribute to their aggressive tumor features, treatment resistance, tumor relapse, and adverse clinical outcomes [36].

The mesenchymal subtype of triple-negative breast cancer is one of the molecular subtypes within TNBC. This subtype is characterized by specific gene expression patterns and features that distinguish it from other TNBC subtypes. Some of the key characteristics of the mesenchymal subtype include EMT (epithelial–mesenchymal transition): Mesenchymal TNBC often shows signs of epithelial–mesenchymal transition. This biological process involves the transformation of cancer cells from an epithelial state (with tight cell–cell adhesion) to a mesenchymal state (with increased motility and invasiveness). EMT is a crucial process in cancer metastasis. EMT allows epithelial cancer cells to acquire mesenchymal characteristics, enabling them to invade, migrate, and eventually form secondary tumors in distant organs.

Also, a hybrid epithelial/mesenchymal (E/M) phenotype refers to cells that exhibit both epithelial and mesenchymal traits simultaneously. This dual characteristic could provide cancer cells with the versatility to navigate through different stages of the metastatic process more effectively than cells committed solely to an epithelial or mesenchymal phenotype [37].

**Figure 2:**
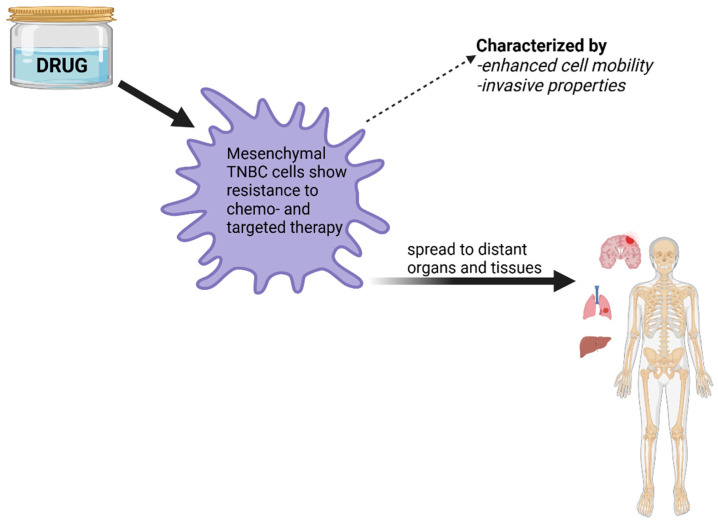
Characteristics of mesenchymal TNBC cells.

Mesenchymal TNBC tumors typically exhibit enhanced cell motility and invasive properties, which can contribute to the cancer’s ability to spread to distant organs and tissues (Figure 2).

Mesenchymal TNBC cells may display resistance to certain chemotherapy regimens and targeted therapies, making treatment more challenging. Also, gene expression patterns within this subtype often reflect mesenchymal characteristics, which can be identified through molecular profiling techniques. Furthermore, due to its invasive properties, the mesenchymal subtype may have a higher likelihood of metastasizing to other parts of the body, such as the lungs, liver, or brain (Figure 2). The mesenchymal subtype of TNBC is characterized by activation of the PI3K pathway [16,27,28].

The luminal androgen receptor (LAR) subtype of TNBC is distinguished by unique gene expression patterns and characteristics that set it apart from other TNBC subtypes. The LAR subtype has some unique characteristics.

**Figure 3:**
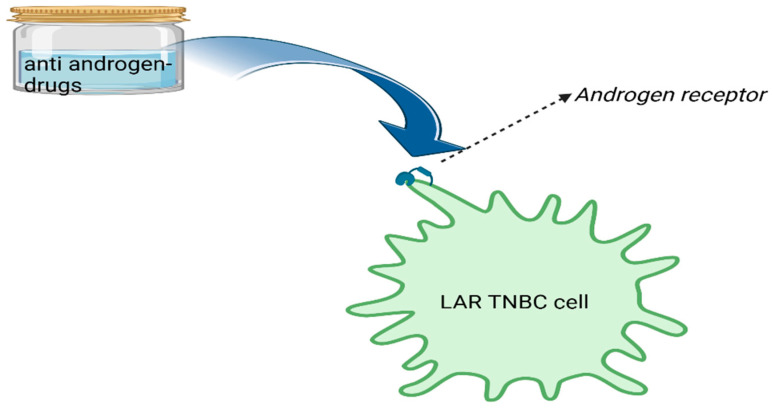
LAR subtype of TNBC with the androgen receptor as a key driver in LAR TNBC and potential therapy options that target the androgen receptor, such as anti-androgen drugs.

The defining feature of the LAR subtype is the expression of the androgen receptor (AR) in the tumor cells. While most breast cancers are driven by estrogen and progesterone receptors, LAR TNBC relies on the androgen receptor for growth and survival. LAR TNBC may exhibit some hormone responsiveness, specifically to androgens (male sex hormones). The androgen receptor significantly influences the growth and progression of this subtype. Because the androgen receptor is a key driver in LAR TNBC, therapies that target the androgen receptor, such as anti-androgen drugs, may be considered as potential treatments. These drugs aim to block the activity of the androgen receptor (Figure 3). LAR TNBC demonstrates a gene expression profile that sets it apart from other TNBC subtypes. In certain respects, it resembles luminal breast cancers, which are typically marked by the presence of hormone receptors. This unique gene expression pattern further underscores the distinct nature of the LAR subtype.

Despite being a subtype of TNBC, LAR TNBC tends to have a better prognosis compared to other TNBC subtypes. Additionally, it may respond differently to various treatment strategies, indicating the importance of accurately identifying and categorizing breast cancer subtypes for tailored therapeutic approaches [16].

The genomic analysis of TNBC reveals that P53 is the most commonly mutated driver gene, followed by PIK3CA. Meanwhile, other genetic alterations such as PTEN, KMT2C, and RB1 are detected at lower frequencies [38,39]. The TP53 gene, responsible for encoding the tumor suppressor protein p53, plays a crucial role in regulating the cell cycle and apoptosis. In TNBC, mutations in TP53 are common and are linked to a poorer prognosis [21,22].

In TNBC, common genomic alterations include gains of 1q, 8q, and 10q; losses of 5q and 8p; amplifications of EGFR and FGFR2; and loss of PTEN [40,41,42,43]. EGFR plays a role in cell proliferation and survival, and its overexpression is associated with a more aggressive disease course. Targeted therapies against EGFR are being explored in TNBC. PTEN is another tumor suppressor gene that negatively regulates the PI3K/AKT signaling pathway, which is crucial for cell proliferation and survival. Loss of PTEN function has been observed in a subset of TNBCs, suggesting potential sensitivity to PI3K/AKT pathway inhibitors [21,22].

**Figure 4:**
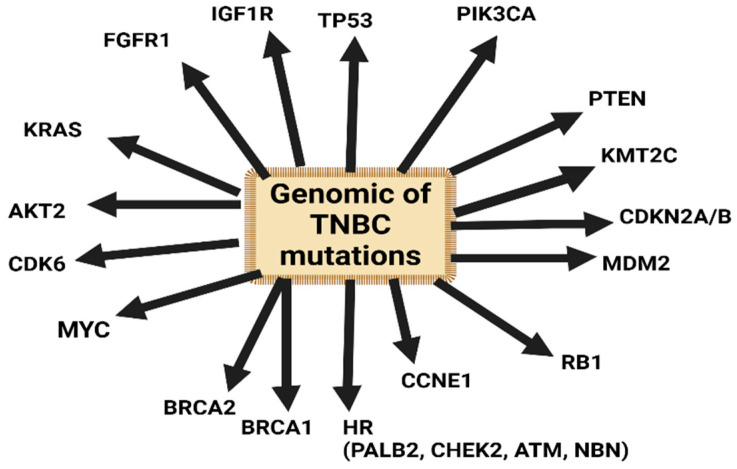
Genomic of TNBC mutations.

The basal-like 1 subtype of TNBC exhibits the highest copy number alterations (CNAs) among TNBC subtypes. It features gains and amplifications in genes like MYC, PIK3CA, CDK6, AKT2, KRAS, FGFR1, IGF1R, CCNE1, and CDKN2A/B, and deletions in BRCA2, PTEN, MDM2, and RB1 (Figure 4) [44].

Defects in double-stranded DNA repair play a key role in the pathogenesis of TNBC, often due to either germline or somatic mutations in BRCA1 and other genes involved in homologous recombination (HR) (Figure 4). The proteins involved in DDR and HR are sensors (like ATM), mediators (like BRCA1 and CHK2), effectors (like BRCA2 and RAD51), or facilitators of the HR pathway (like PALB2 and BRIP1) [45].

In the absence of BRCA1/2 germline mutations, alterations in other specific homologous recombination (HR) genes, such as PALB2, CHEK2, ATM, and NBN, can lead to an increased risk of breast cancer development [46].

Mutations in TP53 and PIK3CA exhibit the highest clonal frequencies, suggesting their significant roles in the early stages of TNBC tumorigenesis (Figure 4) [4,39].

Inherited germline DNA mutations in breast cancer patients frequently result in the loss of function in genes involved in DNA repair and cell-cycle checkpoint activation [47,48].

The susceptibility genes BRCA1 (breast cancer 1) and BRCA2 (breast cancer 2) are critical in the DNA damage response (DDR). The term “gBRCA1/2” refers to germline mutations in these genes. It was observed that when a gBRCA1 mutation is present, it is more likely to lead to the development of triple-negative breast cancer (TNBC) rather than hormone receptor-positive breast cancer (BC). On the other hand, individuals with gBRCA2 mutations tend to develop hormone receptor-positive BC. The impact of somatic BRCA1/2 mutations, which occur later in life and are not inherited, also contributes to breast cancer classification. Somatic mutations in BRCA1 are more commonly associated with basal-like and triple-negative breast cancers, while somatic BRCA2 mutations can lead to various subtypes, including luminal and TNBC. This highlights the importance of considering both germline and somatic mutations in BRCA1/2 when evaluating breast cancer classification and treatment options [47,48,49,50,51,52,53].

PARP inhibitors, particularly PARP-1 inhibitors, play a crucial role in DNA repair and exhibit significant antitumor effects on BRCA1/2-deficient tumors. Among the PARP inhibitors, olaparib and talazoparib are currently approved for treating BRCA-mutated breast cancer, particularly in advanced and metastatic settings. These inhibitors are known for their strong binding affinity to PARP enzymes, leading to effective inhibition of the DNA repair pathways in cancer cells. Niraparib, rucaparib, and veliparib are also PARP inhibitors undergoing clinical trials or development. While they share the same mechanism of action, there are differences in their pharmacokinetics and specific targets within the PARP family. Niraparib and rucaparib, for example, are more selective for PARP-1 and PARP-2, and veliparib shows a broader PARP inhibition profile but with lower potency compared to olaparib and talazoparib. These differences may influence their efficacy and safety profiles, leading to varying clinical outcomes depending on the cancer subtype and patient characteristics [54,55].

Currently approved and effective monotherapy biomarker-targeted oral medications, specifically poly(ADP-ribose) polymerase (PARP) inhibitors, include olaparib and talazoparib. These are designated for use in cases of deleterious or suspected deleterious germline BRCA-mutated, human epidermal growth factor receptor 2 (HER2)-negative breast cancer, particularly for locally advanced and/or metastatic conditions [56].

There are also other PARP inhibitors currently undergoing global clinical trials or in development for the treatment of BC, such as niraparib, rucaparib, and veliparib, and they could show good results administered with platinum-based chemotherapy for HER2-negative, gBRCA-mutated locally advanced or/and metastatic BC [57,58].

Immunotherapies like pembrolizumab, a monoclonal IgG4-K antibody targeting the programmed death receptor 1 (PD-1) pathway, are utilized as monotherapy in patients with TNBC [4,59]. Immunohistochemistry, using specific antibodies for programmed death-ligand 1 (PD-L1), serves as the biomarker for approving PD-1 immune checkpoint inhibitors in breast cancer. Additionally, the combination of atezolizumab—a monoclonal antibody that inhibits the interaction between PD-L1 and PD-1—with chemotherapy has been shown to extend progression-free survival in metastatic PD-L1–positive TNBC patients [60].

In this context atezolizumab and pembrolizumab are approved for use in PD-L1–positive TNBC patients [4].

Despite the known reduced therapy response in triple-negative breast cancer (TNBC), current treatment guidelines recommend combination regimens that include taxane, anthracycline, cyclophosphamide, cisplatin, and fluorouracil. The most commonly used therapy regimens for TNBC in the adjuvant setting include combinations such as taxel/docetaxel with adriamycin and cyclophosphamide (TAC), docetaxel with cyclophosphamide (TC), adriamycin with cyclophosphamide (AC), cyclophosphamide with methotrexate and fluorouracil (CMF), cyclophosphamide with adriamycin and fluorouracil (CAF), and epirubicin with cyclophosphamide, fluorouracil, and paclitaxel/docetaxel (CEF-T) [3,61].

Antibody–drug conjugates (ADCs) are a class of multi-agent drugs designed for targeted delivery of therapeutic small molecules to cancer cells, and they have shown promising results in the treatment of various types of cancer, including triple-negative breast cancer. ADCs enhance the precision and efficacy of cancer treatment by combining the specificity of monoclonal antibodies with the potency of cytotoxic drugs [62,63].

The antibody component specifically targets antigens or receptors that are overexpressed on the surface of cancer cells, ensuring selective binding to these cells.

The linker is a chemical bridge that connects the antibody to the cytotoxic drug. It needs to be stable in circulation but cleavable within the target cell to release the drug.

The cytotoxic drug is a potent chemotherapeutic agent that is highly toxic to cancer cells. Once the ADC is internalized by the cancer cell, the drug is released and exerts its cytotoxic effects [64,65,66].

By identifying these subtypes, and due to the availability of more lines of treatment, the median overall survival of metastatic TNBC has improved from 12 months to around 24 months, as seen in clinical trials [67].

The clinical outcome in triple-negative breast cancer (TNBC) has traditionally been considered less favorable compared to other breast cancer subtypes, mainly due to its aggressive nature, higher likelihood of recurrence, and the absence of targeted therapies like those available for hormone receptor-positive and HER2-positive breast cancers. However, recent advancements in research and treatment strategies are beginning to change the prognosis for some patients with TNBC. Early-stage TNBC has a better prognosis compared to advanced-stage TNBC. The size of the tumor and lymph node involvement at the time of diagnosis significantly influence outcomes. Patients with TNBC who carry BRCA1 or BRCA2 mutations may have a better response to certain chemotherapies and targeted therapies like PARP inhibitors [68]. TNBC is more likely to achieve a pathologic complete response (pCR) to neoadjuvant chemotherapy than other subtypes, which is associated with improved survival outcomes. Chemotherapy remains the cornerstone of treatment for TNBC, with platinum-based agents showing particular efficacy, especially in patients with BRCA mutations. The addition of immune checkpoint inhibitors (e.g., pembrolizumab, atezolizumab) to chemotherapy has shown improved progression-free survival and overall survival in certain patients with advanced TNBC expressing PD-L1 [69]. For patients with germline BRCA mutations, PARP inhibitors like olaparib and talazoparib have shown significant activity, offering a targeted treatment option. A subset of TNBC expresses the androgen receptor, and therapies targeting this receptor are under investigation.

## 3. Challenges

The aggressive biological and clinical characteristics of triple-negative breast cancer (TNBC) lead to more frequent and earlier recurrence compared to other breast cancer types. Research shows that TNBC carries a nearly threefold higher risk of distant recurrence within the first five years after diagnosis, compared to non-TNBC cases [70]. On the contrary, the likelihood of experiencing a relapse after the five-year mark is minimal, at less than 3% [71]. Unlike hormone receptor-positive or HER2-positive breast cancers, the absence of common targets has made developing targeted therapies for TNBC challenging. However, ongoing trials focusing on new targets and treatment combinations are promising. TNBC is a highly heterogeneous disease at the molecular level, which complicates treatment. Identifying and targeting specific molecular subtypes within TNBC could lead to better outcomes [72].

The clinical management of triple-negative breast cancer (TNBC) remains a significant challenge due to its aggressive nature, early recurrence, and lack of specific therapeutic targets. TNBC patients face a nearly threefold higher risk of distant recurrence within the first five years post-diagnosis compared to other breast cancer subtypes. After five years, the risk of recurrence drastically reduces, yet the aggressive course of the disease within the early years highlights the urgent need for effective treatment strategies.

One of the most pressing unmet medical needs in TNBC is the development of targeted therapies. Unlike hormone receptor-positive or HER2-positive breast cancers, TNBC lacks common therapeutic targets, such as estrogen receptors or HER2, limiting the effectiveness of conventional targeted therapies. This absence necessitates the exploration of alternative targets and innovative treatment approaches.

Moreover, the molecular heterogeneity of TNBC adds another layer of complexity to its treatment. TNBC encompasses various molecular subtypes, each with distinct genetic and epigenetic profiles. This diversity complicates the identification of universally effective treatments, as each subtype may respond differently to the same therapeutic approach. Current research is focused on identifying and exploiting specific vulnerabilities within these subtypes, but there is still a considerable gap in translating these findings into clinical practice.

Another critical challenge is the resistance to chemotherapy, which remains the cornerstone of TNBC treatment. While some patients initially respond well to chemotherapy, many experience relapse due to the development of resistance. This resistance is often driven by the presence of cancer stem cells and other adaptive mechanisms within the tumor microenvironment. Addressing chemotherapy resistance requires a better understanding of these mechanisms and the development of combination therapies that can overcome or bypass these resistant pathways.

## 4. Current Treatment Options

Chemotherapy is the primary treatment for TNBC because it does not respond to hormone therapy or HER2-targeted drugs.

Surgical resection remains a cornerstone of treatment for TNBC. The choice between breast-conserving surgery (lumpectomy) and mastectomy depends on the tumor size, location, and patient preference. For many TNBC patients, surgery is typically performed following neoadjuvant chemotherapy to reduce the tumor size, making it possible to opt for breast-conserving surgery. Sentinel lymph node biopsy or axillary lymph node dissection is often conducted to assess the spread of cancer to the lymph nodes, which is an important factor in staging and determining the need for additional treatments.

**Figure 5:**
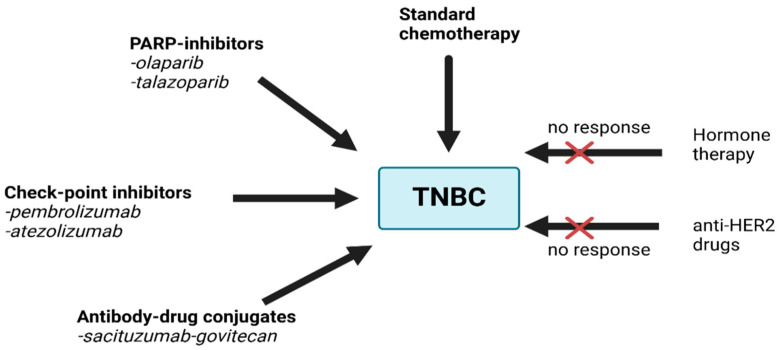
Therapy options for TNBC.

Radiotherapy is commonly recommended after surgery, especially in cases where the tumor was large or there was lymph node involvement. Radiotherapy significantly reduces the risk of local recurrence by targeting residual microscopic disease. It is particularly beneficial in TNBC due to the high recurrence rates associated with this subtype. Evidence suggests that radiotherapy not only reduces local recurrence but also contributes to overall survival benefits in high-risk TNBC patients [72,73]. Recent advancements have led to the development of some targeted therapies for TNBC, such as PARP inhibitors for patients with BRCA mutations and antibody–drug conjugates (like sacituzumab govitecan) [74]. Immunotherapy, such as checkpoint inhibitors, has also shown promise in treating TNBC, particularly in advanced stages. Regular follow-up visits are important to monitor for any signs of recurrence. Supportive care plays a crucial role in managing symptoms and improving the quality of life for those with triple-negative breast cancer (TNBC). This care includes mental health services, physical therapy, and nutritional support. Additionally, genetic testing for mutations such as BRCA1 and BRCA2 is often recommended, as TNBC is more prevalent among women with these genetic alterations. This can also inform treatment strategy and surveillance for other potential cancers (Figure 5, Table 1) [18,21,22].

In the treatment of TNBC, chemotherapy is a critical component because TNBC lacks hormone receptors and HER2 expression, which rules out hormonal therapies and HER2-targeted treatments. Anthracyclines (such as doxorubicin and epirubicin) and taxanes (such as paclitaxel and docetaxel) are commonly used, either alone or in combination. These drugs are effective in both the neoadjuvant setting (before surgery to shrink the tumor) and the adjuvant setting (after surgery to eradicate any remaining cancer cells; Figure 5, Table 1) [75,76].

Regimens like doxorubicin and cyclophosphamide followed by a taxane, such as docetaxel or paclitaxel, are commonly used. Often used in combination with anthracyclines and taxanes, cyclophosphamide can be effective in treating TNBC. It works by interfering with the growth of cancer cells [77]. On the other hand, extracts of plants used in traditional medicine should be considered for further research as a source of bioactive agents with antiproliferative potential, antioxidant activity, and multiple biological properties which show an effective antitumoral effect [78,79,80].

Carboplatin is a platinum-based chemotherapy that is particularly useful in TNBC patients who have BRCA mutations, as these cancers are more responsive to DNA-damaging agents like platinum drugs. Used in combination with carboplatin, gemcitabine can be an option for patients with recurrent or advanced TNBC. This combination is known for its effectiveness in patients who have previously received anthracyclines and taxanes [81,82]. Capecitabine is an oral chemotherapeutic agent that is sometimes used after other chemotherapy treatments have been completed, especially in patients who have residual cancer following neoadjuvant chemotherapy [83]. Although not a traditional chemotherapy, PARP inhibitors like olaparib and talazoparib are used in patients with BRCA mutations. These drugs target cancer cells’ ability to repair their own DNA, leading to cell death (Figure 5, Table 1) [84,85].

Sacituzumab govitecan has been a significant focus of recent research and discussion in the treatment of TNBC. This antibody–drug conjugate targets TROP-2, a cell-surface glycoprotein that is overexpressed in many epithelial cancers, including TNBC. It is linked to a potent chemotherapy agent, SN-38, allowing for direct delivery to cancer cells, thereby potentially reducing side effects associated with non-targeted chemotherapy (Figure 5, Table 1) [74,86].

Combining checkpoint inhibitors like pembrolizumab and atezolizumab with other treatments, including chemotherapy, targeted therapy, or other immunotherapies, is a major focus (Figure 5, Table 1). These combinations aim to enhance the immune system’s response against TNBC [87,88].

Pembrolizumab targets the PD-1 (programmed death receptor-1) on immune cells. PD-1 is a checkpoint protein that cancer cells exploit to avoid being attacked by the body’s immune system. By inhibiting PD-1, pembrolizumab boosts the immune system’s ability to detect and destroy cancer cells. TNBC tends to be more immunogenic than other types of breast cancer, meaning it can induce a stronger immune response. This characteristic makes TNBC a suitable candidate for immunotherapy. Pembrolizumab has shown significant efficacy, especially in TNBC patients whose tumors express high levels of PD-L1 (programmed death-ligand 1), which interacts with PD-1 to suppress immune responses. The KEYNOTE-355 trial was a significant study that evaluated the efficacy of pembrolizumab in combination with chemotherapy in patients with previously untreated, locally recurrent inoperable or metastatic TNBC. This study found that adding pembrolizumab to chemotherapy significantly improved progression-free survival compared to chemotherapy alone, particularly in patients with PD-L1-positive tumors [87,89].

Atezolizumab, like pembrolizumab, is a checkpoint inhibitor used in immunotherapy, but it targets a different checkpoint protein known as PD-L1 (Figure 5, Table 1). Atezolizumab binds to PD-L1, which is expressed on tumor cells and tumor-infiltrating immune cells. PD-L1 usually binds to PD-1 on immune cells, signaling the immune cells to not attack the tumor. By blocking PD-L1, atezolizumab prevents it from interacting with PD-1, thereby enabling the immune system to attack and destroy cancer cells [88]. The IMpassion130 trial was pivotal in demonstrating the efficacy of atezolizumab for TNBC. In this trial, atezolizumab combined with nab-paclitaxel (a taxan) significantly improved progression-free survival in patients with metastatic or unresectable locally advanced TNBC who had not received prior chemotherapy for metastatic disease. The benefits were particularly notable in patients whose tumors expressed PD-L1 [60,90].

Chemotherapy (taxanes, anthracyclines) has been evaluated in multiple trials, including the CALGB 40603 trial. PARP inhibitors, such as olaparib and talazoparib, were assessed in the OlympiAD and EMBRACA trials, respectively, focusing on BRCA-mutated breast cancer. The immune checkpoint inhibitors pembrolizumab and atezolizumab were studied in the KEYNOTE-355 and IMpassion130 trials for metastatic TNBC. The efficacy of the antibody–drug conjugate sacituzumab govitecan was demonstrated in the ASCENT trial for patients with metastatic TNBC [21,22,85,86,87,91].

Reducing the risk of recurrence in TNBC is a crucial focus of treatment strategies, given the aggressive nature and higher recurrence rates associated with this subtype. While integrating the aforementioned therapies is essential, lifestyle modifications and regular follow-up care also play a key role. Adopting a healthy diet, maintaining physical activity, and avoiding risk factors such as smoking can significantly contribute to lowering the risk of recurrence. Consistent monitoring and follow-up care enable the early detection of any recurrence, allowing for timely and effective interventions [92,93,94].

## 5. Conclusions and Future Directions

Current research in the field of TNBC is heavily focused on identifying biomarkers that can serve multiple purposes. Researchers are actively searching for specific molecular and genetic markers that can be targeted with new or existing therapies. These targeted treatments aim to improve the effectiveness of TNBC treatment while minimizing side effects. Identifying biomarkers that can predict the course and outcome of TNBC is essential. This information helps oncologists make informed decisions about the best treatment strategies for individual patients and provides patients with a better understanding of their prognosis.

Determining which patients are likely to respond positively to particular treatments is a key focus of research. By identifying predictive biomarkers, oncologists can tailor treatment plans to maximize benefits and minimize unnecessary exposure to ineffective therapies.

Biomarker research in TNBC includes investigating genetic mutations, gene expression patterns, immune system-related markers, and other factors that can provide valuable information for diagnosis, treatment selection, and patient outcomes. Additionally, these biomarkers are often crucial in the development of personalized and precision medicine approaches, where treatments are customized to each patient’s unique characteristics.

This ongoing research is essential for improving the management and outcomes of TNBC, which is a challenging and heterogeneous form of breast cancer. It underscores the importance of staying up to date with the latest developments in TNBC research, as new biomarkers and targeted therapies continue to emerge and evolve.

Artificial intelligence (AI) can integrate and analyze vast amounts of data from various sources, including genomics, proteomics, imaging, and clinical records. This comprehensive analysis allows for a more detailed understanding of the tumor’s molecular and cellular landscape. AI enhances tumor profiling by enabling more precise, comprehensive, and personalized analysis of tumors, ultimately leading to improved treatment strategies and patient outcomes. This personalized approach not only increases the chances of treatment success but also minimizes the risk of side effects by avoiding unnecessary or ineffective therapies.

By identifying patterns and correlations that might not be apparent to human researchers, AI can help uncover new biomarkers and molecular signatures that are critical for tumor classification and treatment planning. Patients with TNBC should work closely with their healthcare providers to determine the most appropriate treatment strategies based on the latest scientific insights.

## Figures and Tables

**Table 1:** Treatment options and clinical response of TNBC.

Treatment	Overall Response Rate (ORR)	Progression-Free Survival (PFS)	Overall Survival (OS)
Chemotherapy (taxanes, anthracyclines)	30–40%	7–10 months	18–24 months
PARP inhibitors (olaparib, talazoparib)	50–60%	6–8 months	12–16 months
Immune checkpoint inhibitors (pembrolizumab, atezolizumab)	35–45%	7–9 months	18–22 months
Antibody–drug conjugates (sacituzumab govitecan)	35–45%	5–7 months	16–20 months

## Data Availability

Further information concerning the present study is available from the corresponding author upon reasonable request.
